# Urinary albumin creatinine ratio is associated with lipid profile

**DOI:** 10.1038/s41598-024-65037-w

**Published:** 2024-06-27

**Authors:** Sang Won Hwang, Taesic Lee, Young Uh, Jun Young Lee

**Affiliations:** 1 Department of Precision Medicine, Yonsei University Wonju College of Medicine, Wonju, South Korea; 2 Department of Family Medicine, Yonsei University Wonju College of Medicine, Wonju, South Korea; 3 Division of Health Informatics, Institute of Global Health Care and Development, Wonju, South Korea; 4 Department of Laboratory Medicine, Yonsei University Wonju College of Medicine, 20 Ilsan-ro, Wonju, 26426 Republic of Korea; 5 Department of Nephrology, Yonsei University Wonju College of Medicine, 20 Ilsan-ro, Wonju, 26426 Republic of Korea; 6 Transplantation Center, Yonsei University Wonju College of Medicine, Wonju, South Korea; 7 Center of Evidence Based Medicine, Institute of Convergence Science, Yonsei University, Seoul, South Korea

**Keywords:** Glomerular diseases, Cardiovascular diseases, Urinalysis

## Abstract

Moderately elevated albuminuria (30–300 mg/g) is a marker of renal dysfunction and a risk factor of cardiovascular disease. Additionally, several recent studies have reported a relationship between moderately elevated albuminuria and triglyceride (TG) levels. Therefore, we aimed to evaluate the relationship between the urine albumin-to-creatinine ratio (UACR) and total cholesterol (TC), TG, and high-density lipoprotein C (HDL-C) levels. We analyzed data from 19,340 patients from the 2011–2014 and 2019–2020 from the Korea National Health and Nutrition Examination Surveys. Multivariate linear regression analysis showed that the UACR was positively associated with TC and TG levels and negatively associated with HDL-C levels in both Korean women and men. These results were reanalyzed according to the degree of proteinuria (normal, moderately elevated albuminuria, and severely elevated albuminuria (≥ 300 mg/g)). We found a positive relationship between UACR and TC and TG levels, but a negative association with HDL-C levels, except for TC (moderately elevated albuminuria) and HDL-C (moderately elevated albuminuria) in Korean men and TC (severely elevated albuminuria), TG (severely elevated albuminuria), and HDL-C (normal range albuminuria) in Korean women. The correlation between albuminuria and lipid profiles became more evident as albuminuria shift from normal to the severely elevated albuminuria. Thus our multivariate linear regression analysis showed that lipid profiles (TG, TC, and HDL-C levels) were associated with the UACR.

## Introduction

An elevated urine albumin creatinine ratio (UACR) is a marker of renal dysfunction and an independent predictor of cardiovascular disease^1,2^. In addition, several studies have shown that moderately increased albuminuria (UACR < 30 mg/g) within the accepted normal range is associated with higher cardiovascular morbidity and mortality, even in the general population^3,4^. Cardiovascular disease (CVD) is a broad-spectrum disease associated with numerous pathophysiological mechanisms such as inflammation^5^, fatty acid metabolic processes, and cholesterol metabolic process^6^.

Several recent studies have also shown a correlation between triglycerides (TGs) and UACR^7–9^. A Japanese cohort study showed that increased TG levels were an independent risk factor for the development of proteinuria in both men (relative risk [RR], 1.032) and women (RR 1.007)^7^. The Korean National Health and Nutrition Examination Survey (KNHANES) study also showed that TG levels are correlated with the albumin-to-creatinine ratio in adults with hypertension^9^. The Risk Evaluation of cancers in Chinese diabetic Individuals: A longitudinal (REACTION) study also showed that higher TG levels (> 2.3 mmol/L) were associated with the UACR in both men and women^8^. Moreover, treatment of dyslipidemia may be beneficial in reducing albuminuria in patients with chronic kidney disease (CKD)^10^.

Cholesterol and TG levels are influenced by several factors, such as age, sex, diet style, and sleep; therefore, large datasets are required to identify reliable and generalized associations between UACR and lipid profiles. Consequently, we performed three assessments using a nationally representative Korean dataset. First, we conducted a univariate analysis, using Pearson’s and Spearman’s correlation analyses, Followed by multivariate linear regressions to evaluate the relationship between the UACR and total cholesterol (TC), TG, and high-density lipoprotein C (HDL-C). Second, stratification analysis was performed based on the UACR reference values recommended by the Kidney Disease Improving Global Outcome (KDIGO) to compare group-specific patterns of association between the UACR and lipid parameters. Third, we compared the categorized forms of the UACR (eight groups) with blood lipid levels. The UACR groups defined by the KDIGO guidelines were used to identify the associations between albuminuria and dyslipidemia in the multivariate analysis.

## Results

**Figure 1:**
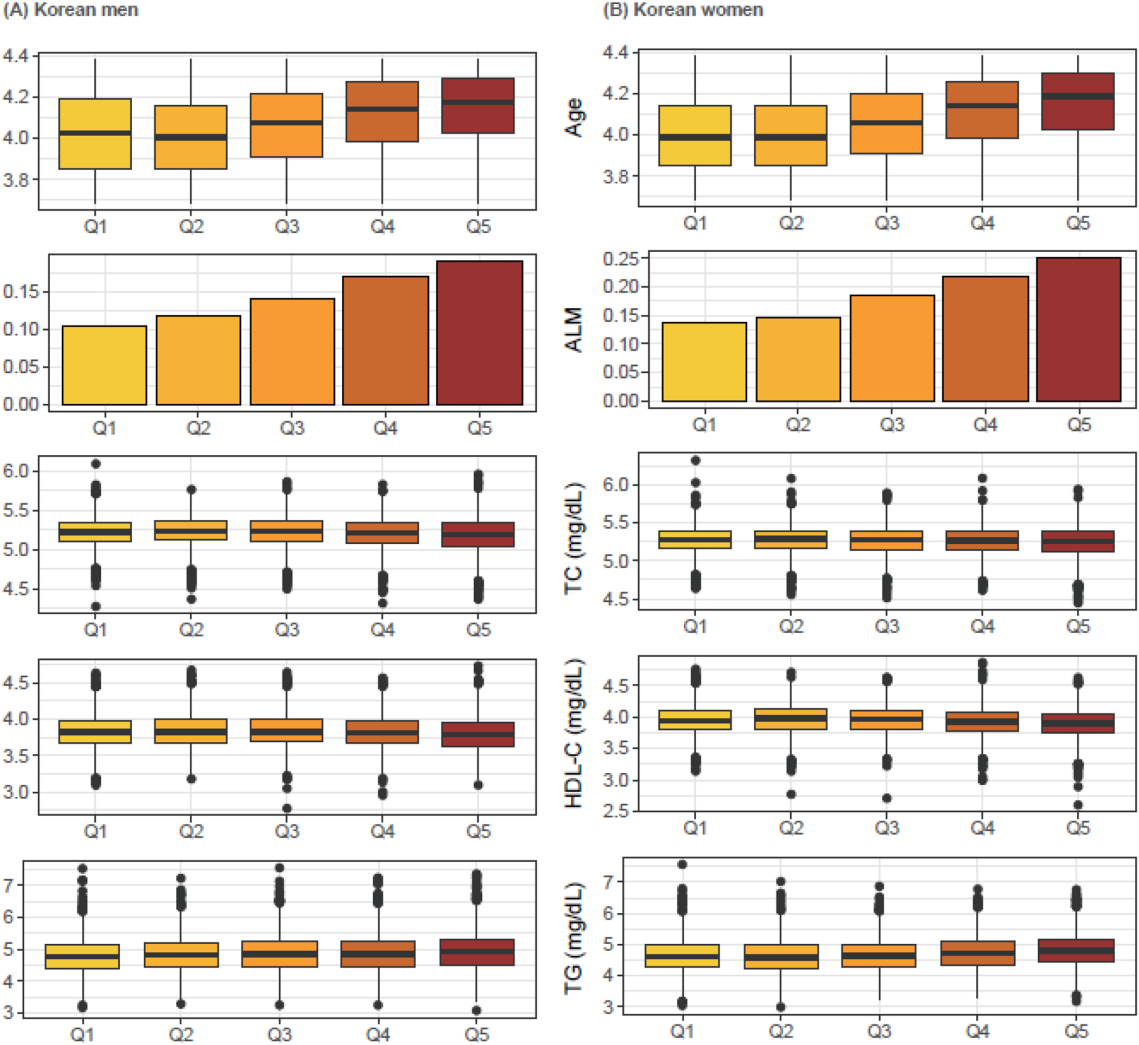
Gender-specific linear trends in lipid profiles according to the UACR (mg/g) quintiles. *ALM* anti-dyslipidemia medication, *HDL-C* high density lipoprotein C (mg/dL), *TC* total cholesterol (mg/dL) *TG* triglyceride (mg/dL), *UACR* urine albumin-to-creatinine ratio (mg/g). *The age on the vertical axis is in log values of years.

All participants were divided into two based groups based on sex: men and women (Fig. S1), to assess sex-specific association pattern between UACR and lipid profiles groups. The UACR was sorted in ascending order and divided into five groups to check for a nonlinear relationship between the degree of albuminuria and lipid profile (Fig. 1 and Figs. S2, S3). Subsequently, as the UACR increased, a monotonically high prevalence of lipid-lowering medication use, and older age was observed in both men and women (Fig. 1). However, the lipid profiles did not exhibit straightforward increasing or decreasing patterns in relation to the UACR; instead, they showed ambiguous and nonlinear relationships that could not be easily explained (Tables S1, S2 and Fig. 1).

**Figure 2:**
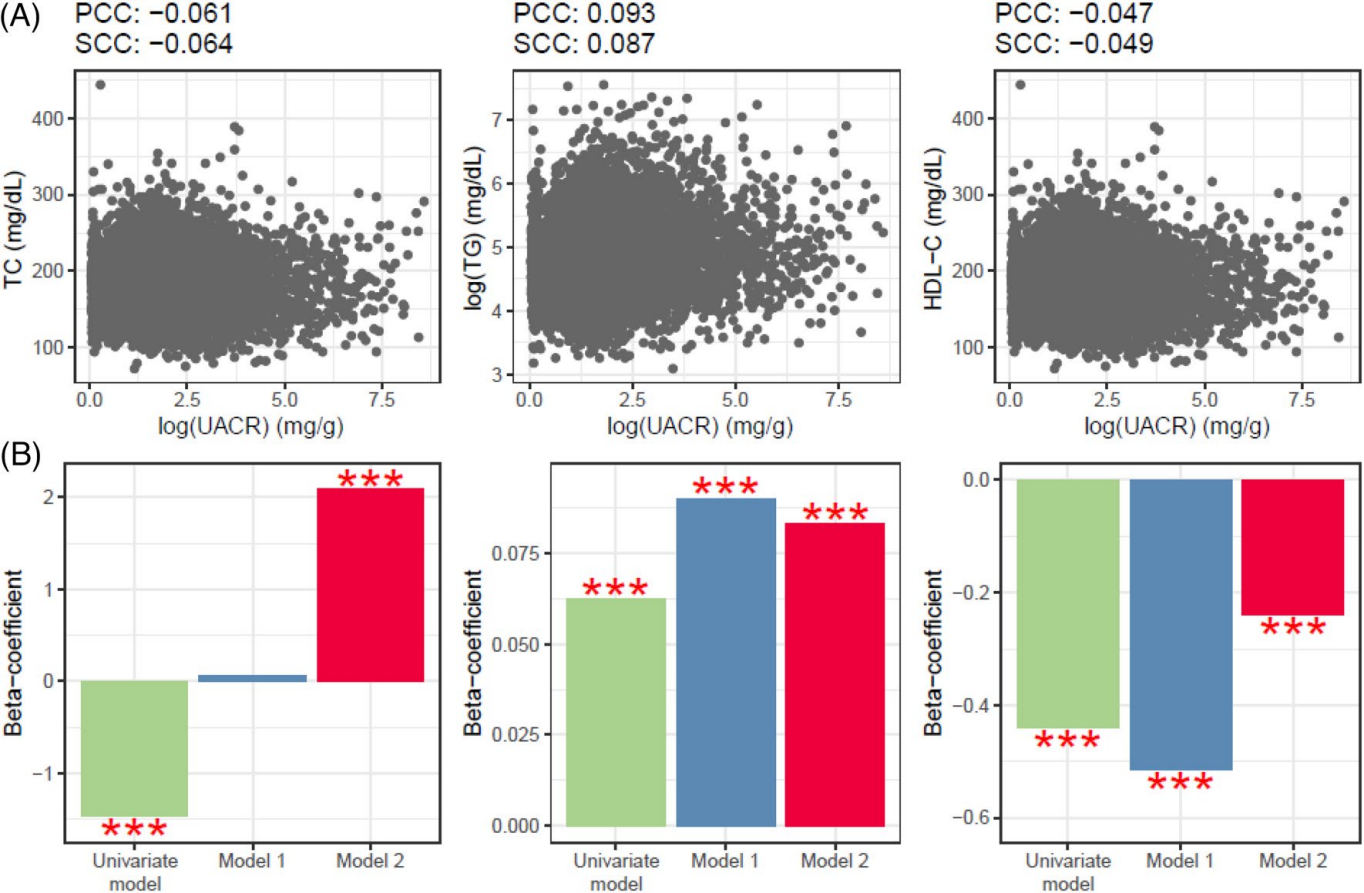
Associational analyses of the UACR and lipid profiles in Korean men. (**A)** Upper scatter plots illustrating the distributions of UACR and lipid profiles, matched with the independent and dependent variables used in the linear regression. For the associations of UACR with three lipid profiles, Pearson’s and Spearman’s correlations (PCC and SCC) were used to calculate the degree of association. (**B**) Linear regression was implemented for association analysis between UACR and lipid profiles. Model 1 included age as the confounder. Model 2 included age, AHM, diabetes, and ALM as covariates. *AHM* antihypertensive medication, *ALM* anti-dyslipidemia medication, *HDL-C* high density lipoprotein C (mg/dL), *PCC* Pearson’s correlation coefficient, *TC* total cholesterol (mg/dL), *TG* triglyceride (mg/dL), *SCC* Spearman’s correlation coefficient, *UACR* urine albumin-to-creatinine ratio (mg/g).

**Figure 3:**
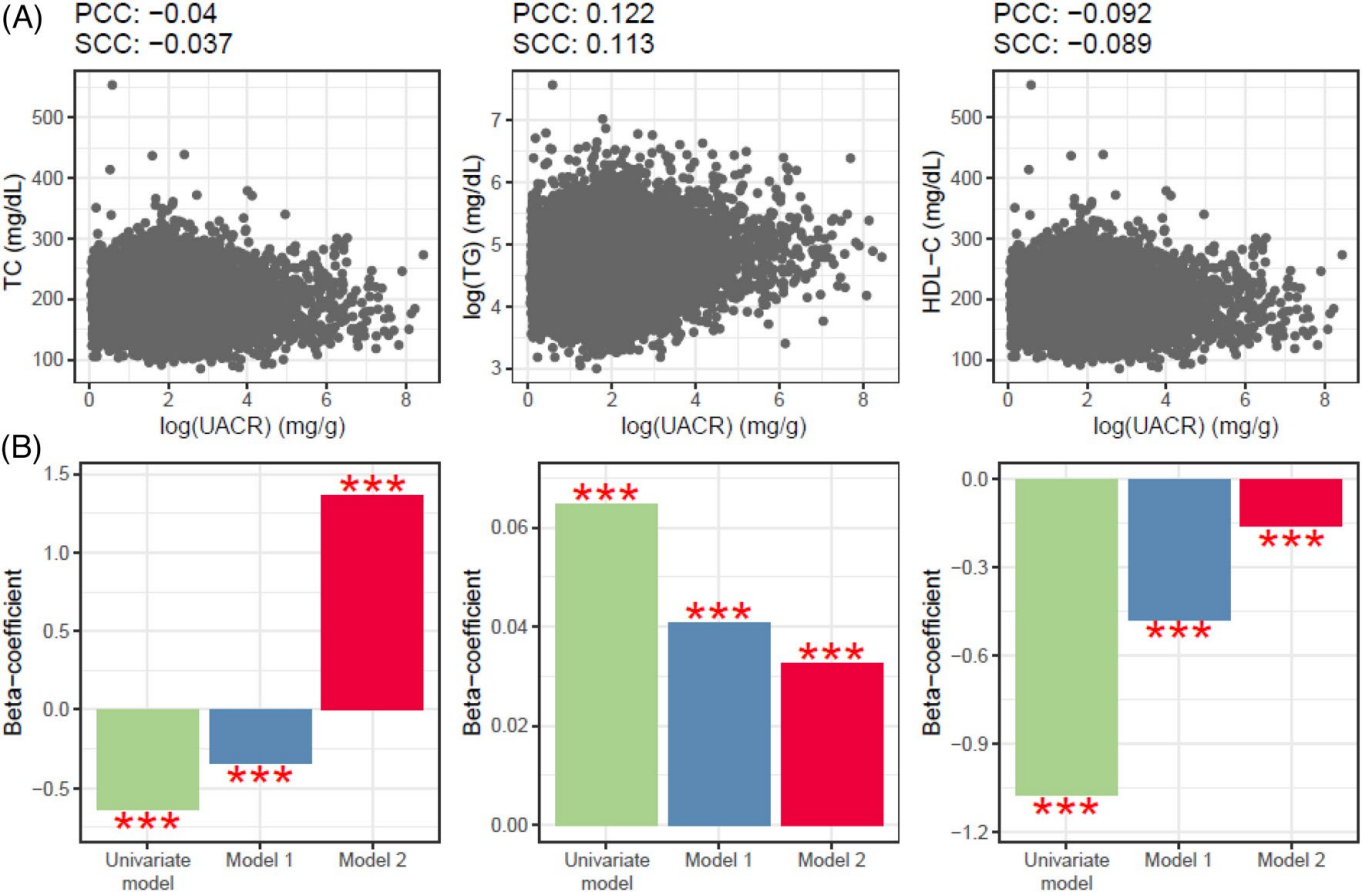
Association between UACR and lipid profiles in Korean women. Two methods (PCC and SCC) were implemented to identify the relationships of UACR level with three lipid profiles. Multivariate linear models were utilized to identify the independent associations of UACR with three lipids. *HDL-C* high-density lipoprotein C (mg/dL), *PCC* Pearson’s correlation coefficient, *TC* total cholesterol (mg/dL), *TG* triglyceride (mg/dL), *SCC* Spearman’s correlation coefficient, *UACR* urine albumin-to-creatinine ratio (mg/g).

Owing to the trend of urinary proteinuria following a long normal distribution (Fig. S1), we performed log transformation of UACR values. Subsequently, we conducted correlation analyses with the lipids (Figs. 2 and 3). The UACR in Korean men was positively correlated with TC based on both Pearson’s and Spearman’s correlation analyses (Fig. 2). Analysis of the association between UACR and TG and HDL-C levels in Korean men showed positive and negative correlations, respectively (Fig. 2A). The association patterns between UACR and lipid profiles in Korean women were consistent with those in Korean men (Fig. 3A).

A multivariate linear regression analysis was conducted to investigate the robust associations between urinary proteinuria and blood lipid profiles (Figs. 2b and 3B). First, we set the log-transformed continuous value of UACR as an independent variable and examined its relationship with TC among Korean men. Proteinuria was found to be negatively correlated with TC, without including covariates, which is consistent with the results of correlation analyses using Pearson’s and Spearman’s correlation analyses. After adding age as a confounding variable, the beta coefficients became positive. In the final linear regression analysis, a significant positive correlation was observed after adjusting for age, antihypertensive medication (AHM), diabetes, and anti-dyslipidemia medication (ALM) (Fig. 2B). TG levels showed a clear positive correlation in the correlation analysis (Fig. 2A) and independent positive associations in both univariate and multivariate analyses (Fig. 2B). HDL-C exhibited a somewhat nonlinear relationship with UACR, but consistently showed a negative correlation in both univariate and multivariate analyses (Fig. 2B). The UACR exhibited an independent positive association with TC and TG and a negative relationship with HDL-C for Korean women in the multivariate analysis (Fig. 3B).

**Figure 4:**
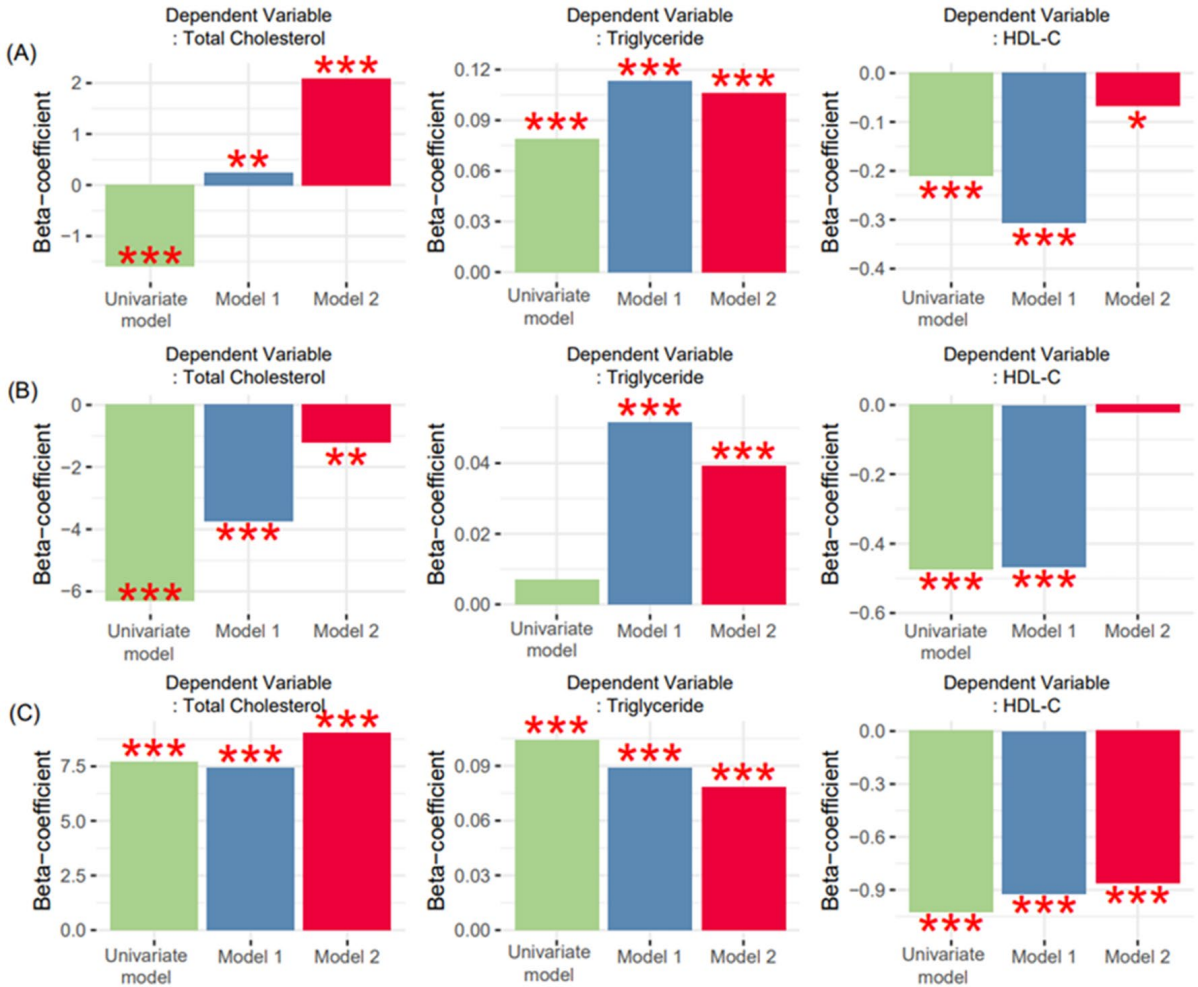
Subgroup association analysis between UACR and lipid profiles according to the KDIGO guidelines^2^ in Korean men. Linear regression was implemented for subgroup association analysis between UACR and lipid profiles. Model 1 included age as the confounder. Model 2 included age, AHM, diabetes, and ALM as covariates. Stratification analysis was conducted according to normal (**A**), moderately elevated albuminuria (**B**), and severely elevated albuminuria (**C**) status. *AHM* antihypertensive medication, *ALM* anti-dyslipidemia medication, *HDL-C* high-density lipoprotein C (mg/dL), *UACR* urine albumin-to-creatinine ratio (mg/g).

**Figure 5:**
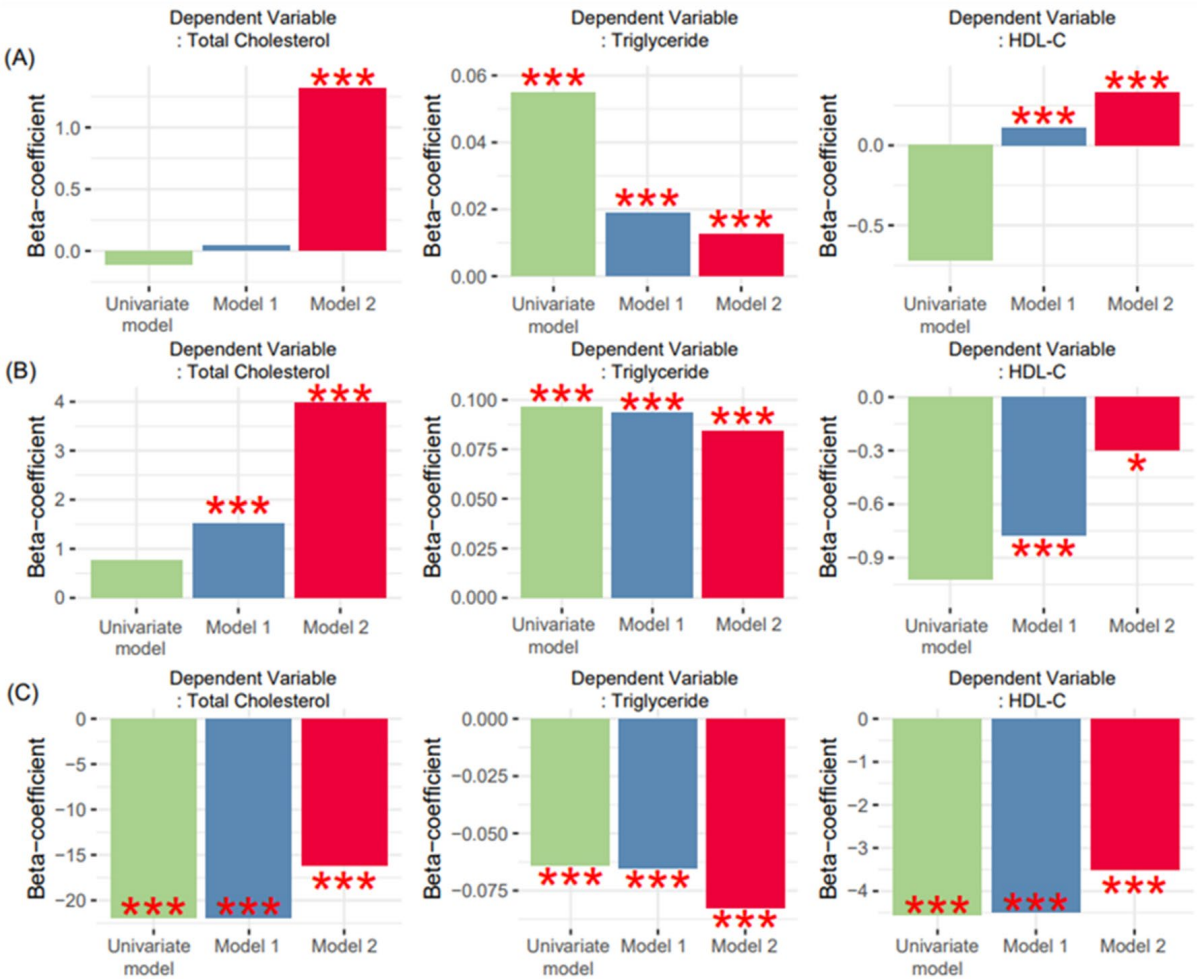
Subgroup association analysis between UACR and lipid profiles according to the KDIGO guidelines^2^ in Korean women. *AHM* antihypertensive medication, *ALM* anti-dyslipidemia medication, *HDL-C* high-density lipoprotein C (mg/dL), *UACR* urine albumin-to-creatinine ratio (mg/g).

Stratified analysis based on the classification of proteinuria according to the KDIGO guidelines^2^ was conducted to determine whether there were significant and consistent patterns of correlation between the continuous variables proteinuria and lipid profiles Figs. S4 and S5). Specifically, we classified the patients into three groups based on thresholds of 30 and 300 mg/g UACR. The UACR showed an independent positive relationship with TC and TG and a negative association with HDL-C in most Korean men, except for TC (UACR: 30–300 mg/g) and HDL-C (UACR: 30–300 mg/g) (Fig. 4 and Fig. S4). These patterns observed in Korean men were mostly replicated in Korean women, except for TC (UACR > 300 mg/g), TG (UACR > 300 mg/g), and HDL-C levels (normal range) (Fig. 5 and Fig. S5).

The UACR were sorted in ascending order and divided into eight groups (Fig. S3). Furthermore, we designated the variables added to Model 3 as confounding variables and conducted a multivariate analysis of the association between the UACR in the eight groups and lipid concentrations. An S-shaped increasing trend was observed for TC in both men and women. A monotonically increasing pattern of TG levels was observed in Korean men. Finally, analysis of HDL-C levels in both men and women revealed, an inverse U-shaped decreasing trend (Figs. S6 and S7).

**Figure 6:**
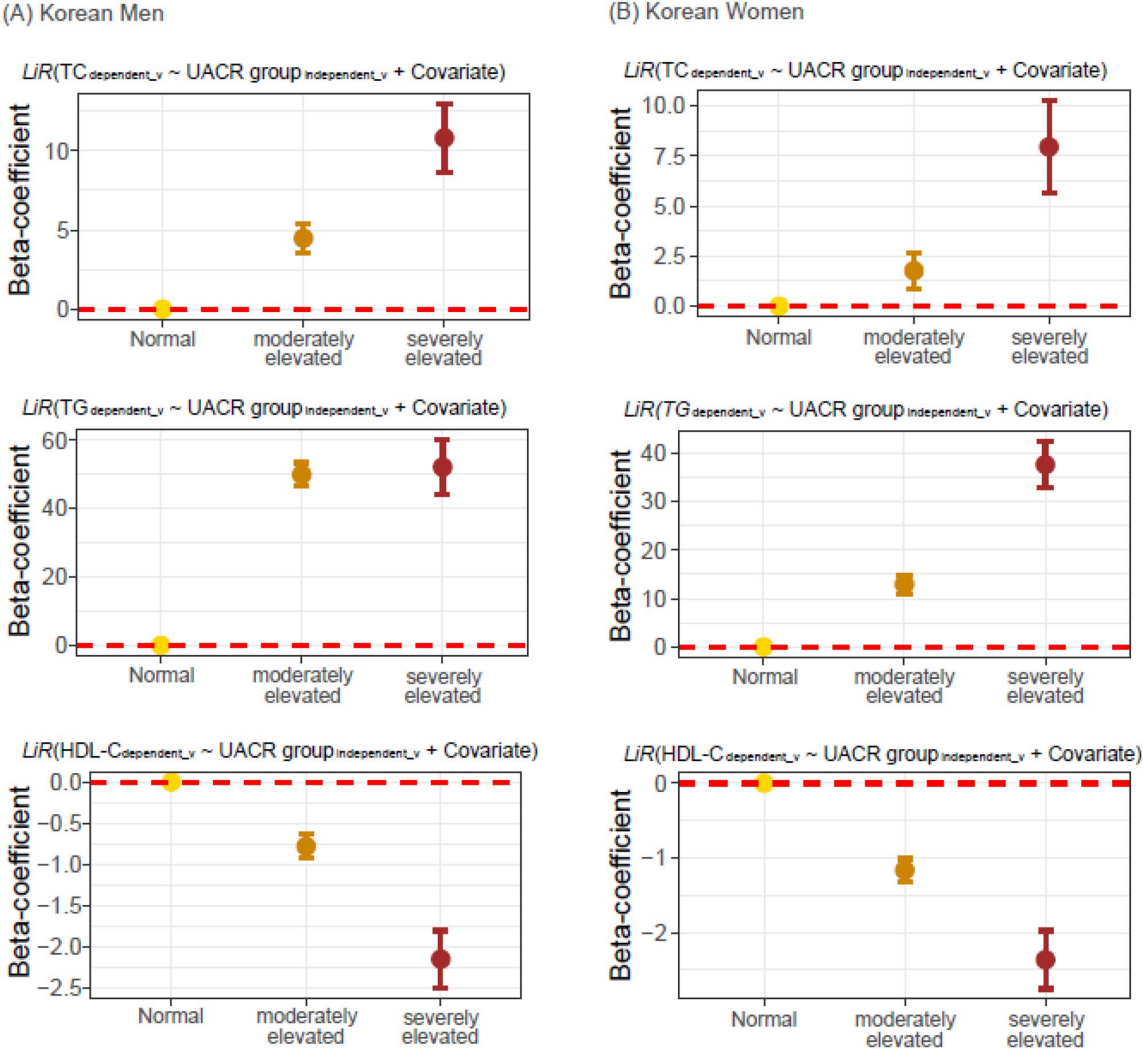
Multivariate linear regression analysis between UACR and lipid profiles in Korean. (**A**) Men, (**B**) Women. *HDL-C* high density lipoprotein-C, *LiR* linear regression, *UACR* urine albumin-to-creatinine ratio, *TG* triglyceride, *TC* total cholesterol.

All UACR cases were categorized into the following three groups according to the KDIGO guidelines^2^: normal, moderately elevated albuminuria (30–300 mg/g), and severely elevated albuminuria (≥ 300 mg/g). Subsequently, multivariate linear regression was performed using the lipid profiles and UACR as the dependent and independent variables, respectively. Monotonic increases in TC and TG levels were observed in both Korean men and women (Fig. 6). In addition, a significant negative relationship was observed between HDL-C levels and UACR in both sexes (Fig. 6).

## Discussion

Our study integrated and analyzed Korean-representative cohorts and demonstrated that the UACR correlated positively with TG and TC and negatively with HDL in both men and women. These results are consistent with stratified analysis. Additionally, we observed the association patterns of the UACR and lipid profiles according to sex and found nonlinear relationships between the UACR and lipid profiles in most lipid profiles; however, a monotonically positive correlation was observed between TG and UACR.

The correlation between TG levels and proteinuria was first reported by Tozawa et al.^7^. They showed that the TG level was a risk factor for the development of proteinuria among 4326 Japanese participants. However, there is limited understanding of the correlation between proteinuria and TG levels as proteinuria was measured using dipstick test. Tien et al. evaluated albuminuria as a categorical variable (normal, moderately elevated, or severe elevated) and TG levels in 2349 Chinese patients with diabetes from two medical centers^11^. Wang et al. evaluated the correlation between TG levels and UACR in 34,569 Chinese participants from eight centers^8^. Moreover, the TG-glucose index recently found to be positively associated with albuminuria in patients with hypertension^12^; however, the UACR measurement units were not unified; therefore, the UACR could not be evaluated as a continuous variable. Conversely, our study analyzed both parameters as continuous variables to provide a more precise analysis.

Protein loss from glomeruli activates several signals through the renal tubules to increase TG production^13^. TG-rich (Apoprotein B) ApoB-containing lipoproteins promote kidney disease progression^14^. Additionally, high TG levels are associated with factor VII and plasminogen activator inhibitor levels, which are in turn associated with intravascular fibrin deposits, thrombosis, and renal fibrosis, resulting in increased urinary albumin excretion^15,16^. Increased TG levels caused by insulin resistance impair glucose metabolism and, trigger oxidative stress and chronic inflammation, which can lead to microvascular permeability and increased glomerular urinary albumin excretion^17,18^. Impaired HDL-C mediated reverse cholesterol transport promotes glomerulosclerosis and tubulointerstitial damage^19^. Moreover, higher HDL-C levels were associated with a reduced rate of microalbuminuria in patients with diabetes^20^. Although the exact mechanism has not yet been identified, we believe that these changes in TG and HDL-C levels affect TC levels, resulting in a positive correlation between UACR and TC levels. Consequently, we observed a strong positive correlation between TG levels and UACR among the lipid profiles.

Therefore, we can consider proteinuria not only as an indicator of kidney dysfunction but also as a marker for dyslipidemia^1^. Additionally, the moderately elevated albuminuria measurement using urine analysis has the advantage of being less invasive and simpler than dyslipidemia measurements using fasting blood tests. The Honolulu Heart Program, which was followed for 27 years, showed that proteinuria, detected by urine dipstick, could independently predict an increased risk of incident CVD^21^. Excessive protein reabsorption in the proximal tubules of the kidney promotes an inflammatory response^22^ and reflects the increased transvascular leakage of various proteins, including lipoproteins^23^. Inflammation elicits local and systemic endothelial dysfunction and CVD^24^.

Nonetheless, this study had some limitations. First, since this was a cross-sectional study, we were unable to establish a causal relationship; only associations between variables could be identified. Second, several parameters (such as past history and prescribed medications) were obtained from the questionnaire that may have led to biased results. Third, we analyzed the KNHANES, which is a previously archived public dataset and does not include information on the detailed type of medication for chronic disease. Therefore, we could not consider the use of medications, such as angiotensin-converting enzyme inhibitors, angiotensin receptor blockers, or sodium glucose transport protein 2 inhibitors, which may reduce albuminuria. Moreover, the KNHANES we used does not include information on the type of lipid-lowering medication but does include a categorical variable for whether a subject is taking medication. Fourth, we used a single urine spot sample to assess UACR rather than a 24-h urine collection. Fifth, because the KNHANES only evaluated low density lipoprotein cholesterol (LDL-C) in hypertriglyceridemia group (TG > 200 mg/dL), it is challenging to identify a generalized relationship between UACR and LDL-C. Moreover, we were unable to obtain data on the factors influencing blood lipid levels, such as thyroid function, body shape, and digestive tract diseases. Nevertheless, we investigated the correlation between albuminuria and lipid profiles in a single representative group using nationally normalized data.

In conclusion, we showed that lipid profiles, especially TG levels, were associated with UACR. This correlation between albuminuria and dyslipidemia indicates that simple spot urine measurement is a highly useful predictor of dyslipidemia and renal function.

## Methods

### Study population

We used data from the KNHANES, a nationwide dataset from Korea. The Division of Chronic Disease Surveillance of the Korea Centers for Disease Control and Prevention in the Ministry of Health and Welfare assesses and monitors nutritional and medical health status through the KNHANES^25,26^. Thus the KNHANES dataset includes nutritional surveys, health examinations, and health interviews that implement a complex multistage probability sample design to obtain nationally representative data. Because the 2015–2018 KNHANES were designed not to evaluate information about UACR, among the approximately 20 datasets containing the clinical information of different participants each year, the KNHANES (2011–2014 and 2019–2020 datasets), including the UACR levels, were used. Participants aged < 40 years and those with missing data were excluded, resulting in 19,340 participants. All KNHANES participants agreed to participate in our study and provided informed consent. The analysis of KNHANES data were performed in compliance with the Declaration of Helsinki. This study was approved by the Institutional Review Board of Wonju Severance Christian Hospital (IRB No. CR321375).

### Covariates

The association between the UACR and CVD or individual predictors in the CVD model has been demonstrated previously^4,27^. Leading groups worldwide have constructed cardiovascular disease prediction models using readily available demographic, physiological, and blood test indicators obtained from clinical settings^28^. Furthermore, advanced models that can better represent complex clinical data have recently been developed through dimensionality transformations such as kernel methods^29,30^. We referenced important studies such as the discovery of the New World by Columbus to select seven variables as covariates.

Six demographic, anthropometric, and laboratory predictors were selected as covariates: age, systolic blood pressure, AHM, current smoking status, and diabetes. Moreover, lipid profiles were considered as dependent variables in linear or logistic regression; therefore, ALMs, such as statins, were included as confounders.

### Statistics

Continuous variables are expressed as means and standard deviations and were analyzed using ANOVAR. The average values of the variables according to the UACR quartiles were used as representative values for each group to test for linear trends in continuous features. Categorical variables are expressed as frequencies and percentages were analyzed using the Chi-squared test. The sampling weights determined by the data constructors were used to estimate the total population represented by the data. After employing the weight values, univariate and multivariate linear regression analyses were used to identify the association between UACR and lipid profiles, such as TG, HDL-C, and TC.

### Supplementary Information

Supplementary Information.

## Data Availability

This study analyzed the data obtained from the KNHANES. The KNHANES database contains publicly available data (https://knhanes.kdca.go.kr/knhanes/sub03/sub03_02_05.do).
