# Supplementary material for: Urinary albumin creatinine ratio is associated with lipid profile
Source: Sci Rep. 2024 Jun 27;14:14870. doi: 10.1038/s41598-024-65037-w (PMC11211387; doi:10.1038/s41598-024-65037-w)
Supplement: Supplementary file 1 — Supplementary Information. [file 41598_2024_65037_MOESM1_ESM.docx]

**Urinary albumin creatinine ratio is associated with lipid profiles**

**(Supplementary Materials)**

Sang Won Hwang^1^, Taesic Lee^2,3^, Young Uh^4^*, Jun Young Lee^5-7^*

^1^ Department of Precision Medicine, Yonsei University Wonju College of Medicine, Wonju, Korea

^2^ Department of Family Medicine, Yonsei University Wonju College of Medicine, Wonju, Korea

^3^ Division of Data Mining and Computational Biology, Regenerative Medicine Research Center, Wonju, Korea

^4^ Department of Laboratory Medicine, Yonsei University Wonju College of Medicine, Wonju, Korea

^5^ Division of Nephrology, Department of Internal Medicine, Yonsei University Wonju College of Medicine, Wonju, Korea

^6^ Transplantation Center, Yonsei University Wonju College of Medicine, Wonju, Korea

^7^ Center of Evidence Based Medicine, Insitute of Convergence Science, Yonsei University, Seoul, Korea

Index

[**Supplementary Figures** 3](#_Toc146030713)

[**Supplementary Figure 1**. Gender-specific distribution of UACR and log(UACR) 3](#_Toc146030714)

[**Supplementary Figure 2**. Subgroup correlations of UACR and lipid profiles according to UACR groups determined by KDIGO guideline in Korean men. 4](#_Toc146030715)

[**Supplementary Figure 3**. Subgroup correlations of UACR and lipid profiles according to UACR groups determined by KDIGO guideline in Korean women. 5](#_Toc146030716)

[**Supplementary Figure 4**. Associational patterns of lipid profiles with eight groups of lipid profiles in Korean men. 6](#_Toc146030717)

[**Supplementary Figure 5**. Associational patterns of lipid profiles with eight groups of lipid profiles in Korean women. 7](#_Toc146030718)

[**Supplementary Tables** 8](#_Toc146030719)

[**Supplementary Table 1**. General characteristics of Korean men according to UARC level. 8](#_Toc146030720)

**Supplementary Figures**

**Supplementary Figure 1**. Gender-specific distribution of UACR and log(UACR)


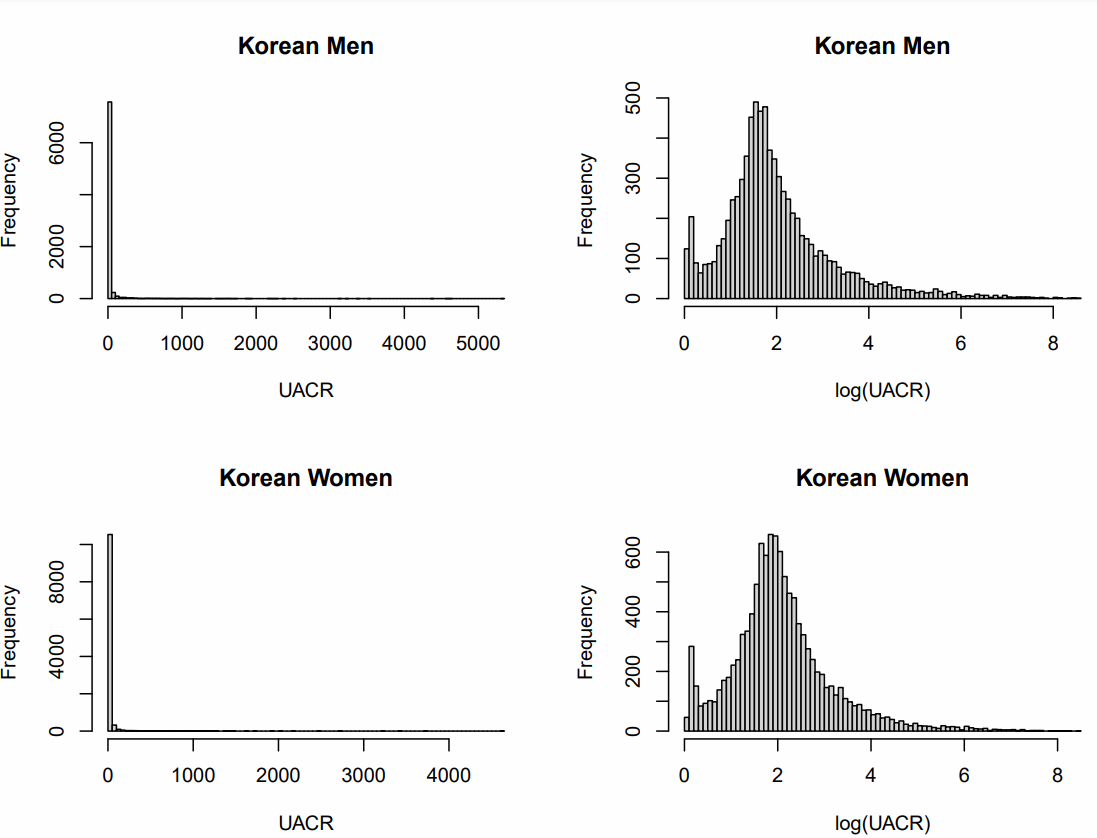


**Supplementary Figure 2**. Subgroup correlations of UACR and lipid profiles according to UACR groups determined by KDIGO guideline in Korean men.


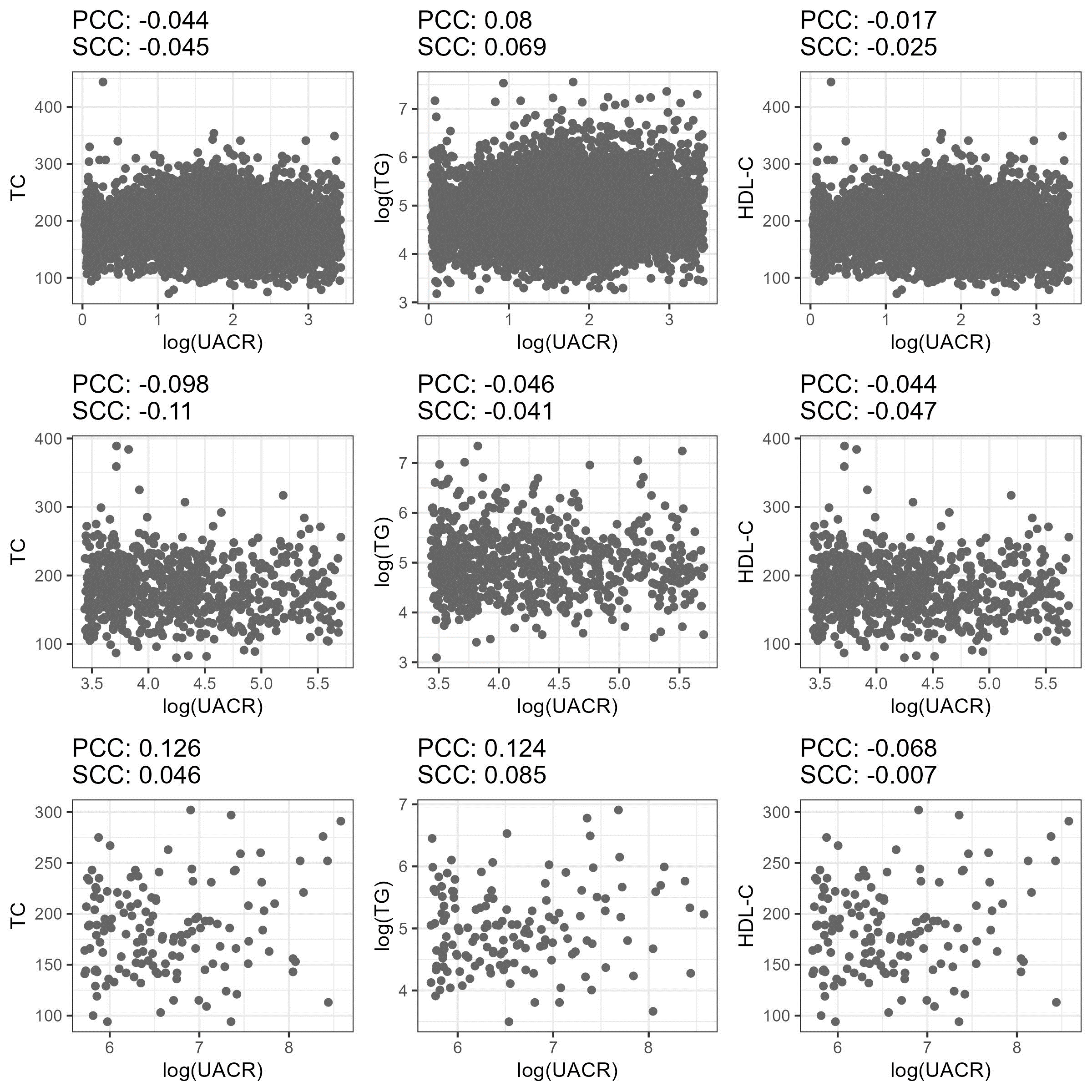


**Supplementary Figure 3**. Subgroup correlations of UACR and lipid profiles according to UACR groups determined by KDIGO guideline in Korean women.


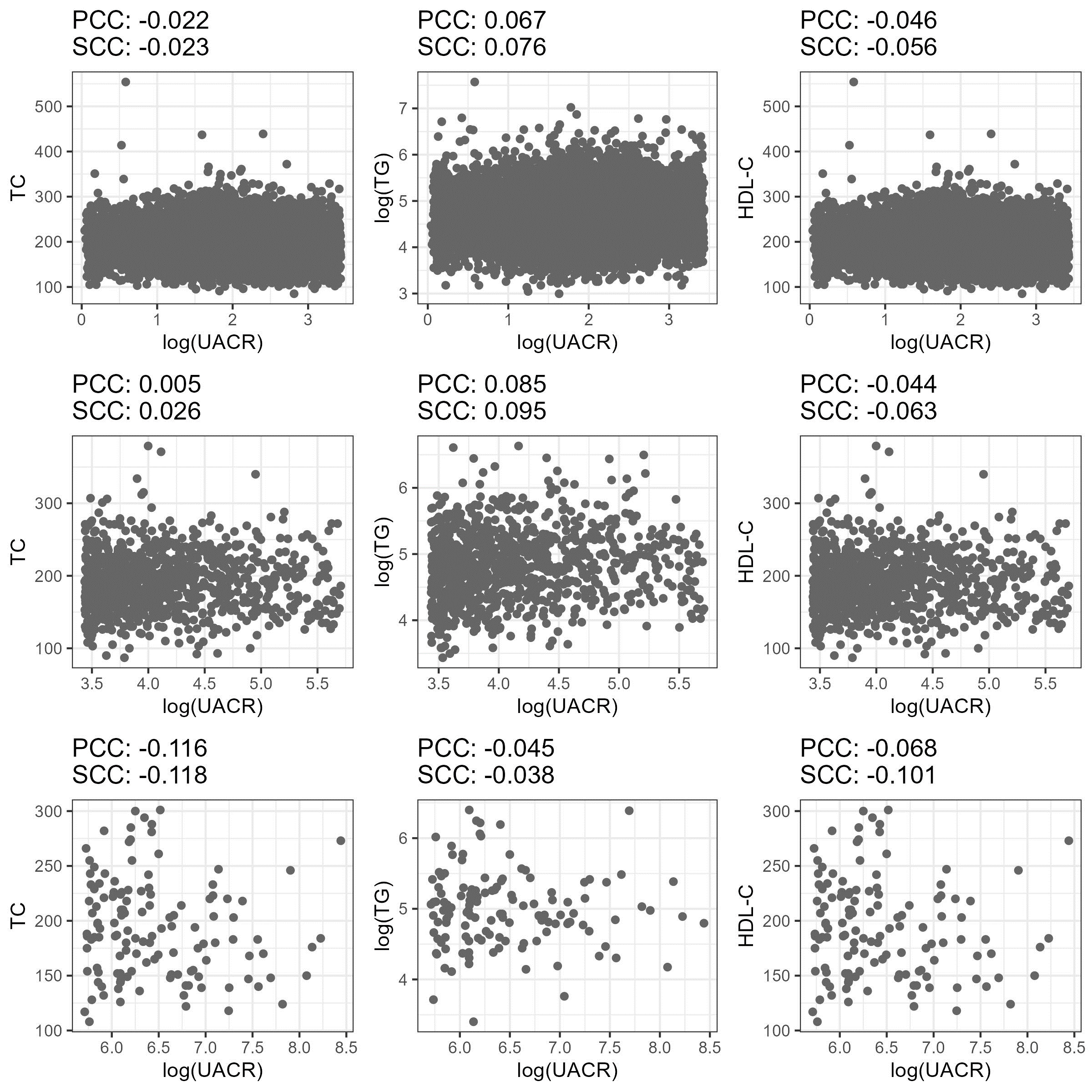


**Supplementary Figure 4**. Associational patterns of lipid profiles with eight groups of lipid profiles in Korean men.

The Associations were calculated based on multivariate linear regression. The eight groups of lipid profiles were determined based on even arrangement of ascending order of UACR.


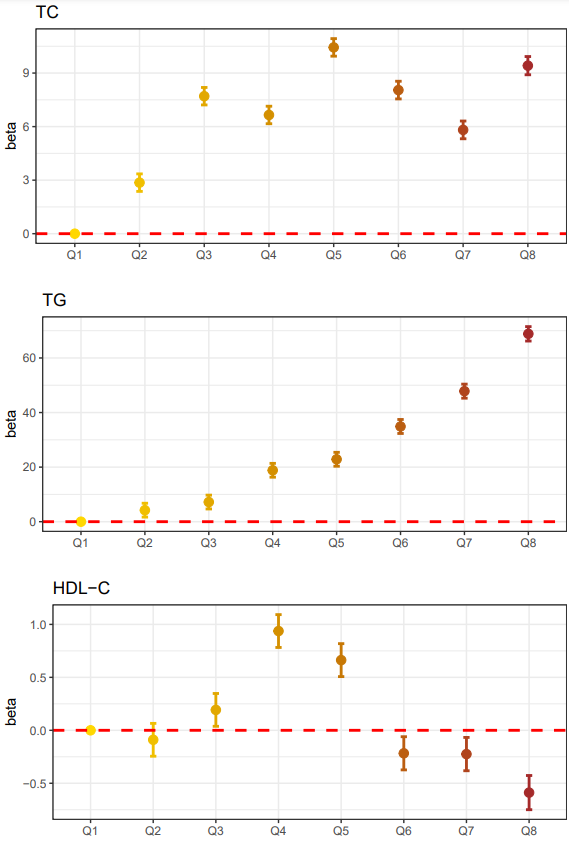


**Supplementary Figure 5**. Associational patterns of lipid profiles with eight groups of lipid profiles in Korean women.

The Associations were calculated based on multivariate linear regression. The eight groups of lipid profiles were determined based on even arrangement of ascending order of UACR.


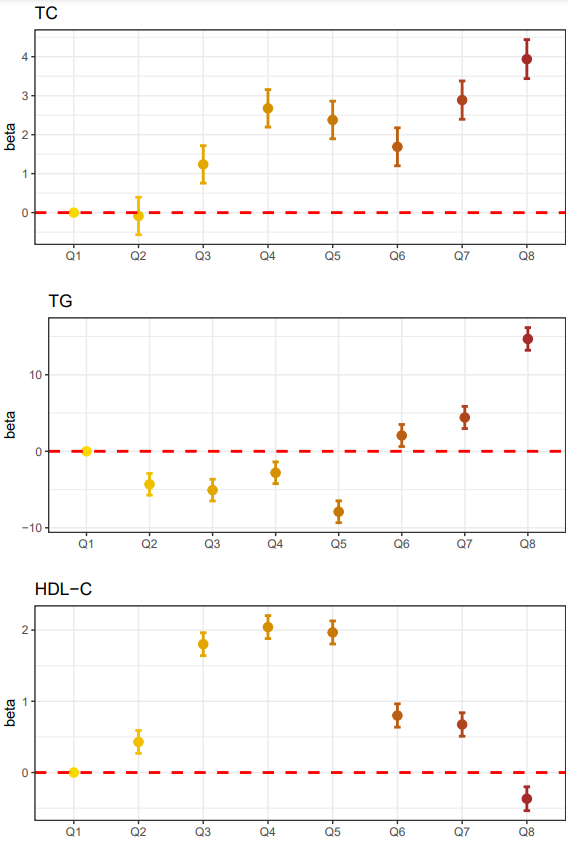


**Supplementary Tables**

**Supplementary Table 1**. General characteristics of Korean men according to UARC level.

|  | Q1 (n=1,631) | Q2 (n=1,630) | Q3 (n=1,630) | Q4 (n=1,630) | Q5 (n=1,631) | p |
| --- | --- | --- | --- | --- | --- | --- |
| Age (years) | 56.9 ± 0.28 | 56.2 ± 0.27 | 58.9 ± 0.28 | 62.6 ± 0.28 | 63.9 ± 0.27 | <0.001 |
| SBP (mmHg) | 119.2 ± 0.36 | 119.4 ± 0.34 | 122.1 ± 0.36 | 125.5 ± 0.38 | 130.7 ± 0.43 | <0.001 |
| HTN medication | 328 (20.1) | 341 (20.9) | 449 (27.5) | 585 (35.9) | 784 (48.1) | <0.001 |
| Diabetes | 104 (6.4) | 122 (7.5) | 159 (9.8) | 287 (17.6) | 513 (31.5) | <0.001 |
| Smoking | 530 (32.5) | 558 (34.2) | 510 (31.3) | 509 (31.2) | 574 (35.2) | 0.054 |
| Total cholesterol | 188.4 ± 0.9 | 190.2 ± 0.9 | 190 ± 0.9 | 185.2 ± 0.9 | 182.4 ± 1.0 | <0.001 |
| HDL | 47.1 ± 0.2 | 47.6 ± 0.2 | 47.6 ± 0.3 | 46.8 ± 0.3 | 46.2 ± 0.3 | <0.001 |
| Urine albumin | 0.2 ± 0.0 | 0.5 ± 0.0 | 0.8 ± 0.0 | 1.4 ± 0.0 | 15.6 ± 1.0 | <0.001 |
| Urine Creatinine | 139.1 ± 1.6 | 164.8 ± 1.9 | 162.3 ± 1.8 | 153.8 ± 2.0 | 140.8 ± 1.8 | <0.001 |
| UACR | 1.1 ± 0.0 | 3.1 ± 0.0 | 4.9± 0.016 | 9.0 ± 0.1 | 134.6 ± 9.5 | <0.001 |

HDL, high density lipoprotein; HTN, hypertension; SBP, systolic blood pressure; UACR, urine albumin protein ratio

**Supplementary Table2.** General characteristics of Korean women according to UARC level (Korean women)

|  | Q1 (n=2,238) | Q2 (n=2,237) | Q3 (n=2,238) | Q4 (n=2,237) | Q5 (n=2,238) | p |
| --- | --- | --- | --- | --- | --- | --- |
| Age (years) | 55.6 ± 0.2 | 55.6 ± 0.2 | 58.4 ± 0.2 | 62.3 ± 0.2 | 64.6 ± 0.2 | <0.001 |
| SBP (mmHg) | 116.1 ± 0.3 | 116.6 ± 0.3 | 120.3 ± 0.4 | 125 ± 0.4 | 131.8 ± 0.4 | <0.001 |
| HTN medication | 412 (18.4) | 482 (21.5) | 565 (25.2) | 794 (35.5) | 1128 (50.4) | <0.001 |
| Diabetes | 122 (5.5) | 136 (6.1) | 182 (8.1) | 294 (13.1) | 517 (23.1) | <0.001 |
| Smoking | 77 (3.4) | 85 (3.8) | 81 (3.6) | 90 (4) | 79 (3.5) | 0.851 |
| Total cholesterol | 197.5 ± 0.8 | 198.1 ± 0.8 | 197.7 ± 0.8 | 196.2 ± 0.8 | 193.4 ± 0.85 | <0.001 |
| HDL | 52.9 ± 0.3 | 54 ± 0.3 | 53.9 ± 0.3 | 51.9 ± 0.3 | 50.4 ± 0.3 | <0.001 |
| Urine albumin | 0.1 ± 0.0 | 0.5 ± 0.0 | 0.7 ± 0.0 | 1.2 ± 0.0 | 7.3 ± 0.4 | <0.001 |
| Urine Creatinine | 97.7 ± 1.1 | 122.8 ± 1.4 | 119.3 ± 1.4 | 116.5 ± 1.4 | 102 ± 1.3 | <0.001 |
| UACR | 1.4 ± 0.0 | 3.923 ± 0.0 | 6.1 ± 0.0 | 10.0 ± 0.0 | 89.1 ± 5.3 | <0.001 |

HDL, high density lipoprotein; HTN, hypertension; SBP, systolic blood pressure; UACR, urine albumin protein ratio
